# A report on the research leadership and scientific writing training organized in Yaounde by the Clinical Research Education, Networking and Consultancy (CRENC) and the IeDEA-Cameroon team

**DOI:** 10.11604/pamj.2018.29.195.14593

**Published:** 2018-04-02

**Authors:** Anastase Dzudie, Epie Njume, Roger Ajeh, Eric Walter Yone-Pefura, Bonghaseh Divine, Adebola Adedimeji, Kathryn Anastos

**Affiliations:** 1 Clinical Research Education, Networking and Consultancy, Douala, Cameroon; 2 Faculty of Health Sciences, University of Buea, Buea, Cameroon; 3 Faculty of Medicine and Biomedical Sciences, University of Yaounde 1, Yaounde Cameroon; 4 Jamot Hospital, Yaounde, Cameroon; 5 Albert Einstein College of Medicine, New York, USA

**Keywords:** Leadership, scientific writing, training, IeDEA

## Abstract

Sub-Saharan Africa has the largest number of individuals leaving with HIV/AIDS. However, much is still unknown as regards HIV/AIDS treatment outcomes in resource-constrained settings. The Cameroon Central Africa International Epidemiologic Databases to Evaluate AIDS-Cameroon (Cameroon CA-IeDEA) collaboration is a unique opportunity to explore long-term outcomes from a large HIV cohort and generate massive data that can show trends, inform HIV care and provide insight on the way forward. Given the lack of research capacity in the country, the need for high impact training that can leverage Cameroon CA-IeDEA has never been more acute.

## Introduction

Scientific productivity and research in Africa for Africa is limited and unequally distributed across the continent. Capacity strengthening, developing skills in scientific writing [1, 2] and leadership training [3], have been postulated to mitigate this trend. It is on this premise that the Clinical Research Education, Networking and Consultancy (CRENC) through Dr. Anastase Dzudie (Principal investigator (PI) for the Cameroon CA-IeDEA project), organised this two-day training on *Leadership and Scientific Writing* under the auspices of the Albert Einstein College of Medicine through its tenured Professors Kathryn Anastos and Adebola Adedimeji (PI and associate PI of the Central African Region IeDEA project respectively). The International Epidemiologic Database to Evaluate AIDS (IeDEA) research study is funded by the United States National Institutes of Health (NIH) and the National Institute of Allergy and Infectious Diseases. The CA-IeDEA study is part of the IeDEA global research consortium, involving seven regions: West, East, Central and Southern Africa; South and North America; and Asia and Pacific. The CA-IeDEA is funded through Albert Einstein College of Medicine, USA. The overall goal of the IeDEA study is to use secondary clinical, laboratory and epidemiologic data from HIV-infected patients in various regions to answer HIV/AIDS and other related co-morbidities research questions that cannot be answered with existing individual cohorts in each country. The IeDEA research consortium makes it possible to generalize study findings to wider settings and populations, with the goal of improving HIV care across countries in the world. The Cameroon IeDEA study obtained its first ethical clearance from the Cameroon National Ethics Committee in March 2013 under the contract research organisation (CRO) Research for Development International (R4DI). The implementation of the study was transferred from R4DI to the current CRO-CRENC in 2016.

## Conference outlines and outcomes

Participants included the project site teams (IeDEA-CRENC) for the 3 project sites in Cameroon (Bamenda Regional Hospital, Jamot Hospital Yaounde and Limbe Regional Hospital), the central project management team (IeDEA-CRENC), representatives from the host facility- Jamot Hospital, and members of the CRENC. The training opened with a presentation by Dr Dzudie (Cameroon) on the CRENC. His talk featured the CRENC's role in building a research culture from the undergraduate through post-doctoral levels and ensuring growth via reciprocal mentor-mentee relationship. The talk was followed by panel discussions during which Prof Anastos (USA) shared her personal experience as a clinician-researcher. She emphasised that the journey from the research question to eventual publication was not easy or smooth and straightforward, but was fraught with challenges, journal rejection, and that the key was to persevere to reach a set goal. Prof Anastos then gave a brief history of Einstein programs in Central Africa, specifically Rwanda where working mainly with non-governmental organisations, civil society organisations and the government, the Einstein consortium (funded by several grants) improved vulnerable populations' access to HIV diagnosis and treatment. There was also extensive research capacity and infrastructure building including research methods, data management and statistical analysis, publication and grants management. The goal of which was to inform clinical care as regards effectiveness of anti-retroviral therapy (ART), nutritional status and its effect on disease progression and response to therapy, the impact of comorbidities (posttraumatic stress disorder, depression) and sequelae of violence on adherence to and effectiveness of ART, and the prevalence/incidence of cervical dysplasia and human papilloma virus (through the Rwanda/Einstein Consortium in HIV/Human Papilloma Virus malignancies). Prof Anastos also gave a brief overview of the National Institute of Health-funded IeDEA study; the 7 global IeDEA regions, the 4 IeDEA regions in Africa, and the Central African IeDEA run by the Albert Einstein College of medicine. She dwelled on the Central African IeDEA, which involves Burundi, Cameroon, Democratic Republic of Congo, Republic of Congo and Rwanda. She shared the successes that have been realised in Rwanda and hoped they be replicated in the other Central Africa IeDEA countries, taking into account the peculiarities in the different countries, especially Cameroon. As regards Cameroon, she talked about the history of the Cameroon IeDEA, challenges with the former CRO and appreciated the current CRO for its efforts in ensuring the smooth running of the study thus far. She also shared her personal experiences as a student, her early career as a physician working among vulnerable populations, and then as a clinical researcher. The manner in which she handled her successes and not giving up whenever she failed was a source of inspiration and encouragement to the participants.

The second day of the training began with a recap of the previous day's activities and this was followed by a lecture on collaborative leadership by Prof Anastos. The central theme of the presentation was that a collaborative leader is one who engages team members to buy into a vision, keeps them focused, working together in a mission to achieve a common goal. And that trust and conflict management were the ingredients to building such a successful collaborative leadership. This was followed by a lecture on management by Prof Adedimeji. From the beginning of the lecture, it was clear that effective leadership and management are interwoven and have similar themes. However, understanding managerial competencies and properly distributing them across the various levels of management, and effective communication were essential to being a good manager and thus an effective leader. He also elaborated stakeholders' management and communication as core elements in effective managerial and leadership competence. Dr. Dzudie presented on needs assessment for capacity building. He began by pointing out the fact that research capacity in Africa was rudimentary, at best mirrored by the very few publications especially when compared to other regions [4]. Also, the few publications are disproportionately distributed, from a few number of countries [5]. Poor research culture and/or training, absence or inadequate infrastructures, absence of effective leadership and lack or insufficient funding are the root causes of this low productivity [3]. He went further to outline 3 levels;- individual, organisational and institutional at which action can be taken to address this deficit and illustrated the CRENC's model of intervention to address this gap. Continuous research education and leadership training at the individual level, solid management structure with clear and concise standard operating procedures in all areas at the organisational level and implementing of a legal framework, partnerships and networking at the institutional level were the three actions levels described.

**Outcomes:** The lecture was followed by a panel discussion during which participants benefited from the vast experiences of the facilitators and tested their understanding of the various modules of the training. The training officially ended with a resolution by Cameroon CA-IeDEA staff to create a research committee to formulate and answer at least one research question from the IeDEA study by November, ahead of the all African IeDEA meeting in Rwanda.

## Conclusion

Research and scientific productivity in sub-Saharan Africa is much lower than would be expected for a region with a high burden of HIV-AIDS, and compared to other regions mainly due to inadequate training, poor leadership and infrastructure, and funding. The CRENC CA-IeDEA team has identified research leadership and scientific writing training as an integral component to leverage research capacity building in general and to enhance quantity and quality research output in Africa, for Africa.

## Competing interests

The authors declare no competing interest.
